# Supplementary material for: Impact of mixed-species forest plantations on soil mycobiota community structure and diversity in the Congolese coastal plains
Source: PLoS One. 2024 Oct 10;19(10):e0311781. doi: 10.1371/journal.pone.0311781 (PMC11469602; doi:10.1371/journal.pone.0311781)
Supplement: S1 Table — (DOCX) [file pone.0311781.s001.docx]

|  |  |  | Faith’s phylogenetic diversity | Pielou Evenness |
| --- | --- | --- | --- | --- |
| Kruskal-Wallis (all groups) |  |  | 0.3327 | **0.0005** |
|  |  |  |  |  |
| Kruskal-Wallis (pairwise) | 100% Acacia (n=9) | 100% Euca (n=9) | 0.1223 | **0.0017** |
|  |  | 50A50E (near Euca) (n=9) | 0.8946 | 0.4529 |
|  |  | 50A50E (near Acacia) (n=8) | 0.6304 | 0.1489 |
|  | 100% Euca (n=9) | 50A50E (near Euca) (n=9) | 0.1023 | **0.0009** |
|  |  | 50A50E (near Acacia) (n=8) | 0.3359 | **0.0015** |
|  | 50A50E (near Euca) (n=9) | 50A50E (near Acacia) (n=8) | 0.5637 | 0.1019 |

100% Acacia = Acacia monoculture stands; 100% Euca = Eucalyptus monoculture stands; 50A50E (near Euca) = soil sampled near Eucalyptus of mixed stands; 50A50E (near Acacia) = soil sampled near acacia of mixed stands.
