# Supplementary material for: Impact of mixed-species forest plantations on soil mycobiota community structure and diversity in the Congolese coastal plains
Source: PLoS One. 2024 Oct 10;19(10):e0311781. doi: 10.1371/journal.pone.0311781 (PMC11469602; doi:10.1371/journal.pone.0311781)
Supplement: S2 Table — (DOCX) [file pone.0311781.s002.docx]

| Samples |  | pseudo-F | p-value |
| --- | --- | --- | --- |
| 100% Acacia | 100% Euca | **3.049633** | **0.001** |
|  | 50A50E (near Euca) | 1.722965 | **0.002** |
|  | 50A50E (near Acacia) | 1.703588 | **0.006** |
| 100% Euca | 50A50E (near Euca) | 0.859575 | **0.002** |
|  | 50A50E (near Acacia) | 1.486676 | **0.002** |
| 50A50E (near Euca) | 50A50E (near Acacia) | 1.768169 | 0.843 |

100% Acacia = Acacia monoculture stands; 100% Euca = Eucalyptus monoculture stands; 50A50E (near Euca) = soil sampled near Eucalyptus of mixed stands; 50A50E (near Acacia) = soil sampled near Acacia of mixed stands.
