# Supplementary material for: Impact of mixed-species forest plantations on soil mycobiota community structure and diversity in the Congolese coastal plains
Source: PLoS One. 2024 Oct 10;19(10):e0311781. doi: 10.1371/journal.pone.0311781 (PMC11469602; doi:10.1371/journal.pone.0311781)
Supplement: Attachment — Submitted filename: PONE-D-24-15839_Reviewercomments_F.docx [file pone.0311781.s003.docx]

\# Reviewer

The manuscript deals with the investigation of the fungal community in pure and mixed plantations of eucalypt and acacia in the Congo. The information is relevant and unprecedented for the region. The information was analyzed by different statistical techniques and is presented in figures. However, my perception is that the manuscript needs a broad revision to simplify the message and direct the discussion to the results of the present study. In addition, the authors cite different articles developed apparently in the same experimental device, but the relationship between the results is not very evident in the discussion.

L17-18. Include the full scientific name of the tree species.

L 29-33: It's confusing. Which planting model(s) provide the most benefits?

L 33-35 This phrase makes more sense at the beginning of the results (L19).

L 35-37. It's confusing. Soil attributes were also related to monospecific plantations. It is unclear what positive effect it has on the resilience of the ecosystem.

L47. It could include citations for this statement or specify which regions this occurs. My perception is that interest in mycobiota has increased due to advances in laboratory techniques. The interest is not evident in the conversion of natural forests to planted forests in all tropical regions. In Brazil, for example, planted forests replace areas previously used for agriculture and livestock in most cases. One interest that seems to be growing is to understand the feedback between plant diversity (e.g., by converting pure forest plantations to mixed plantations) and microbial communities. Perhaps mentioning something about this would help link to the next paragraph.

L 54. Why Eucalyptus and/or Acacia? It needs a minimum of two species.

L 71-74. My perception is that this sentence is confusing and perhaps in the wrong place. Was this territorial extension occupied only by mixed plantations of eucalypt and acacia?

L 79. What type of forest management?

L80-81. Could you specify which forest management and type of stand the authors are comparing? Could you include references to work done elsewhere to support your hypothesis?

L 82. Don't composition and diversity have the same meaning in the sentence? Wouldn't it be more appropriate to replace it with community structure and diversity?

L 89-91. Average air temperature is an important piece of information as well.

L 92-93. The first sentence is more in line with the first paragraph.

L94. The description of the experimental device seems confusing to me. Are there three replications with monospecific acacia plantations? And just one stand with eucalypt monoculture? Is it just a stand with mixed planting? Would this be a complete randomized block design?

L 98-100. Wasn't there a new mixed planting? It's confusing because it seems that there is no mixed planting for data collection.

L 105. What is the length of the transect? 10 m (similar to the width of the sampling plot)? If that's the case, it seems odd that the sampling points are only 0.7 m apart from each other.

L110-115. Routine soil fertility practice is to use a 2 mm sieve. Also, it seems confusing to me the way he mixed the simple samples to form the composite samples. In addition, it is unclear how long the sampling and molecular analysis took place, and how the samples were transported from the field to the lab.

L117. The elemental analyzer provides the total content of each element. So, it would be appropriate to include the term Total (e.g. Total C, N, and S...).

L129-130. Specify how these samples were distributed among treatments.

L 152. Wouldn't a parametric test (ANOVA, Tuckey, etc.) be more appropriate since the data come from a randomized experimental device? Ditto for Spearman's correlation, which could be replaced by Pearson. Has the normality of the data been tested?

L 186-189. I don't remember reading that described in the methodology that the samples were collected near eucalypt or acacia trees in mixed plantations.

L 189-192. Both sentences should be in the methodology.

L 192-196. It seems to me that the association of several statistical techniques to present the results is confusing. I miss a greater link between the first sentence (PCA) and the second sentence (Table S2).

L 217. Report this proportion of unidentified phyla.

L231. Authors should avoid single-sentence paragraphs. In addition, the S does not seem to have been the most important element in explaining the differences between the treatments. Perhaps it would be better to separate the information into positive and negative correlations, as this leads to different impacts.

L252-254. Does this mean that only P and N contributed significantly? C and S, or C/N, C/P, N/P... Have they not contributed significantly?

L263. Have any tests been conducted to assess this? Was the difference statistically significant?

L 265. The legend of this figure should be improved. There are color variations and colors that have no meaning (such as the shades of red, and orange in 50E:50A near euca).

L271-272. The authors could more adequately explain these results, given the size of the figure, and the different patterns colors, and connections. How are these connections made?

L 276-284. This paragraph is more appropriate in the introduction than in the discussion.

L293-294. The difference between generalists and rare ones was small. The sum of the two can represent up to 73% of mycobiota. One question would be whether specialist species have a particular ecological function.

L 296. Eucalyptus in this case should be italicized.

L296-297. That sentence seems confusing to me. If the number of species is greater in pure plantations, wouldn't they be more resilient to environmental stresses? Would increasing the diversity of fungi in theory be more beneficial, or not?

L297-300. The authors could better explain these ratios since they were not presented at the M&M.

L 300-306. I have doubts whether the eucalypt litter would necessarily be more recalcitrant than that of the acacia. The crucial difference seems to me to be the N content, which is higher in acacia leaves. But acacia leaves take longer to decompose than eucalypt leaves.

L 311. “.. potential associations with function (40,41)”. Quais functions?

L 311-313. Although there are no studies on the diversity of the fungal community in forest plantations of eucalyptus, acacia, and the mixture of the two species in the Congo, this hypothesis is somewhat presumptive to be accepted. The authors cite studies showing that microbial communities are to some extent modulated by the species planted on the site (e.g. 4, 17, 18). Perhaps the authors could go beyond that. For example, the authors mention in the introduction that fungi are important for carbon sequestration in the ecosystem, forest productivity, etc. Wouldn't it be possible to make that kind of relationship? For example, the abstract states: "Higher Faith's phylogenetic diversity and Evenness' was found in Eucalyptus monoculture relative to stands containing Acacia." Did this result in improved or resistance in some productivity parameters (e.g. growth, tree biomass, survival, litterfall?

L 314. I don't recall reading a highlight about endomycorrhizae versus ectomycorrhizae in M&M and outcomes. I think it's important.

L 360-364. The authors highlight important functions of fungi in ecosystems in these sentences but have little to do with the results. For example, it could explain the strong positive correlation between the total C (and C/P) of the soil and the Myxotrichaceae family.

L322-323. This statement is confusing. What the authors mean by "may have been compensated." Is it related to the growth/survival of trees?

L 335-336. The authors could include some explanation for the lack of correlation of this phylum (Ascomycota) with the total C contents.

L337-350. Were the percentage differences statistically significant? In Figure 2A, there do not seem to be major differences, but %100E and %100A show extreme values, while mixed plantings show intermediate percentages for some phyla and families. Perhaps it is more appropriate to concentrate the discussion on the phyla and families that were correlated with soil fertility parameters.

L 356-358. It is unclear what impact these issues (afforestation and oil production) will have. What would be the potential impact of this? (e.g. for productivity, ecosystem services of plantations?).

L360-361. This statement would make more sense if information about fungal communities in the reference area as native ecosystems were presented.

L369-371. Authors could include references to support this claim. It seems to me inappropriate to assume this based on correlations.

L 379-382. This statement seems limited to me. What would be the anthropic activity? Wouldn't it be better to discuss it from the perspective of diversity and its effects?

L382-383. How strong was this correlation? Was it always positive or negative? It seems to me that there is a lack of discussion in this regard.

L383-386. My perception is that there is a lack of a more evident link between human activities and soil alteration. In addition, the last sentence does not seem to be consistent with Figure 3.

L387-399. That paragraph is a conclusion, but the information could be better discussed in the discussion.
